# Combination of *Lactobacillus plantarum* and *Saccharomyces cerevisiae* DV10 as Starter Culture to Produce Mango Slurry: Microbiological, Chemical Parameters and Antioxidant Activity

**DOI:** 10.3390/molecules24234349

**Published:** 2019-11-28

**Authors:** Xiaofan Jin, Wenxue Chen, Haiming Chen, Weijun Chen, Qiuping Zhong

**Affiliations:** 1 College of Food Science and Engineering, Hainan University, Haikou 570228, China; jinxiaofan412@163.com (X.J.); hnchwx@163.com (W.C.); hmchen168@126.com (H.C.); 2 Chunguang Agro-Product Processing Institute, Wenchang 571333, China

**Keywords:** probiotic product, mango slurry, *Lactobacillus plantarum*, *Saccharomyces cerevisiae*, volatile compounds, antioxidant power

## Abstract

The aim of this study was to develop a nondairy fermented product based on mango slurry. *Lactobacillus plantarum* and *Saccharomyces cerevisiae* DV10 were used as starter cultures in single and co-cultivations. The microbial populations and metabolites produced during mango slurry fermentation were investigated. At the end of all fermentations, the bacterial populations were higher than 6.0 log CFU/mL. Lactic acid was the main organic acid produced, achieving up to 6.12 g/L after 24 h in co-culture with *L. plantarum* and *S. cerevisiae* DV10. Volatile compounds were determined after 24 h of fermentation, the co-cultures of *L. plantarum* and *S. cerevisiae* DV10 could decrease terpenes and produce alcohols and esters. The co-cultivations obtained the most total phenolics as well as showed the strongest 2,2′-azinobis-(3-ethylbenzothiazoline-6-sulfonate) (ABTS) radical scavenging activity, ferric-reducing antioxidant power (FRAP) and low-density lipoprotein (LDL) oxidation inhibition. Hence, a high-bioactivity probiotic product was successfully obtained via mango slurry fermentation inoculated with a co-culture of *L. plantarum* and *S. cerevisiae* DV10.

## 1. Introduction

Probiotics microorganisms benefit the health of the host by improving the intestinal microbiota composition [1]. At present, there is growing interest in the development of new functional foods and the use of probiotic microorganisms in healthy diets [2]. Using probiotic microorganisms to ferment beverages can improve the nutritional value and sensory properties of these beverages [3]. The use of probiotic microorganisms in dairy beverages has been widely promoted [2]. However, high fat, high cholesterol, lactose intolerance, milk allergy and vegetarian growth have prompted study in the field of nondairy probiotic products [2]. Fruit are rich in nutrients and contain sugars to support the growth of probiotic microorganisms. Therefore, fruit are considered to be an ideal substrate for the development of nondairy probiotic beverages [4].

Mangoes (*Mangifera indica* L.) are rich in nutrients, sweet and delicious and are popular among the public. It is among the most important tropical fruit in the world [5]. The production and international trade of mangoes are gradually expanding [6]. Mangoes contain a variety of biologically active compounds, such as polyphenols, carotenoids, vitamins and dietary fiber, and have nutritional and antioxidant properties [7]. Due to its juicy and sweet taste, mangoes are known as the king of fruit and are widely used by consumers throughout the world at various maturity stages. Besides the fresh fruit, mangoes are processed into various products such as slices, juices, nectars and jams [8]. As far as we know, a study of a mixed fermentation mango slurry using lactic acid bacteria (LAB) and yeast has not been reported. Mixed culture fermentations provide complex growth patterns that can also considerably affect the functional and organoleptic properties of food [9]. Hence, the aim of this work was to develop a novel mango slurry product fermented by using probiotic strain *Lactobacillus plantarum* association with yeast *Saccharomyces cerevisiae* DV10. Microbial growth during fermentation, pH and the contents of organic acids, volatile compounds and total phenols before and after fermentation were determined. Furthermore, antioxidant capacity and copper-induced low-density lipoprotein (LDL) oxidation were also evaluated.

## 2. Results and Discussion

### 2.1. Microbial Growth Performance During Mango Slurry Fermentation

**Figure 1:**
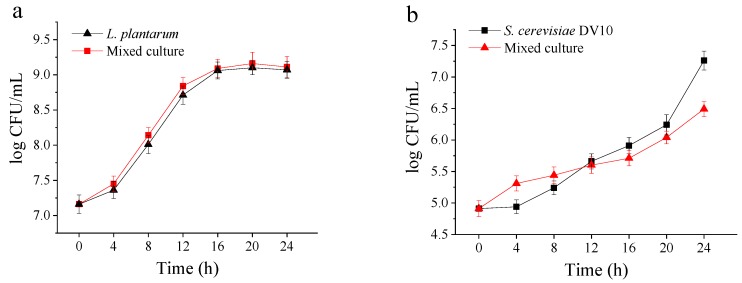
Microbial populations during single and co-culture fermentations of mango slurry. (**a**) Populations of *Lactobacillus plantarum* in single and mixed culture fermentations. (**b**) Populations of *Saccharomyces cerevisiae* DV10 in single and mixed culture fermentations.

Figure 1 shows the microbial growth of *S. cerevisiae* DV10 and *L. plantarum* during single and co-culture fermentations of mango pulp. The growth rate of *L. plantarum* was not affected by the presence of *S. cerevisiae* DV10 in the mixed culture and remained similar to that of the *L. plantarum* pure culture (Figure 1A). Other studies have used a combination of LAB and yeast to ferment oats; the observed behavior of LAB was similar to our results [10]. There was no significant (*p* > 0.05) difference in the bacterial population in the single or co-culture assays (9.07 log CFU/mL and 9.11 log CFU/mL, respectively) at 24 h of fermentation, and the bacterial populations of both approaches were significantly (*p* < 0.05) higher than the initial population (7.16 log CFU/mL). High viable counts were important to get the lower pH, growth of contaminants was prevented [11].

Regarding *S. cerevisiae* DV10, as shown in Figure 1B, the initial population was 4.91 log CFU/mL. The population in single and co-culture fermentations significantly increased (*p* < 0.05) within 24 h, reaching 7.26 log CFU/mL and 6.49 log CFU/mL. The populations obtained at 24 h of fermentation were higher than that required for probiotic products (6–7 log CFU/mL) to have a healthy effect on the gastrointestinal tract when consumed [10]. After 24 h of fermentation, the population of *S. cerevisiae* DV10 in single culture was higher than that of the mixed culture. This may be due to the inhibition of *S. cerevisiae* DV10 by cyclic peptides and phenyl lactic acid produced by *L. plantarum* growth and metabolism. Co-cultured organisms may compete for nutrients or may produce metabolites that stimulate or inhibit each other’s growth [9]. The results show that the combination of *L. plantarum* and *S. cerevisiae* DV10 can be appropriate for fermenting mango pulp.

### 2.2. Quality Parameters

The pH of mango pulp fermented with *L. plantarum* (single and co-culture) rapidly decreased from 4.12 to 3.55 over 24 h (Table 1). The pH of the single *S. cerevisiae* DV10 fermented mango pulp was observed to decrease from 4.12 to 3.98. This may have been related to the higher amount of lactic acid in the fermentation of *L. plantarum*. After 24 h of fermentation, the total soluble solids (TSS) of *S. cerevisiae* DV10 (single and co-culture) fermented mango pulp decreased from 21.6 to 20.2° Brix, and the reducing sugar content decreased from 2.24 to 1.97 g/L. TSS in the mango pulp fermented with single *L. plantarum* decreased from 21.6 to 21.2° Brix, and the reducing sugar content decreased from 2.24 to 2.13 g/L. After 24 h of fermentation, the *S. cerevisiae* DV10 (single and co-culture) cultures were significantly lower (*p* < 0.05) in TSS and reducing sugar content than the single *L. plantarum* culture (Table 1). 

### 2.3. Changes in TPC, ABTS and FRAP

Total phenolic content (TPC) in the unfermented mango pulp were 75.87 mg GAE/100 mL (Table 1). TPC of the single *S. cerevisiae* DV10, single *L. plantarum* and mixed fermentation mango pulp were 79.41, 86.59 and 89.25 GAE/100 mL at 24 h of fermentation, respectively. Long-term consumption of plant polyphenol-rich diets can prevent the development of cancer, cardiovascular disease and diabetes [12]. In addition, the lower pH of the *L. plantarum* fermented juice was beneficial to the stability of polyphenols because they were auto-oxidized as the pH increases [13]. Some studies have found that fermenting juice with LAB can alleviate the degradation of macropolymeric phenolic substances and increase the total phenolics [14,15]. TPC in the co-cultivation of *S. cerevisiae* DV10 and *L. plantarum* was the highest over 24 h.

After 24 h of fermentation, the ABTS free radical scavenging ability of the single *S. cerevisiae* DV10, single *L. plantarum* and mixed fermentation mango pulp significantly increased from 10.43% to 12.72%, 15.29% and 16.11%, respectively (Table 1). Some scholars have also obtained similar results after fermenting cultures with LAB or *S. cerevisiae* [16,17]. Other reports have found that the increase in ABTS free radical scavenging was because of an increase in TPC [18]. After 24 h of fermentation, the mango pulp fermented with the co-cultivation of *S. cerevisiae* DV10 and *L. plantarum* showed the strongest ABTS radical scavenging ability.

As shown in Table 1, the ferric-reducing antioxidant power (FRAP) of mango pulp fermented with single *S. cerevisiae* DV10 slightly increased from 1.11 mM FeSO_4_ to 1.16 mM FeSO_4_ over 24 h. The FRAP of the single *L. plantarum* and mixed fermentation mango pulp significantly (*p* < 0.05) increased to 1.47 and 1.49 mM FeSO4, respectively. A previous study has found that FRAP was increased after fermentation with *L. plantarum* compared to unfermented samples [16]. Some scholars believed that FRAP may be related to the TPC, and the presence of phenolic compounds in a sample extract leads to a reduction in the TPTZ-Fe^3+^ complex to the TPTZ-Fe^2+^ form [19]. Therefore, the higher TPC in the assays containing *L. plantarum* may help to increase FRAP. After 24 h of fermentation, co-cultivation of *L. plantarum* and *S. cerevisiae* DV10 showed the highest FRAP. 

### 2.4. Inhibition of LDL Oxidation

**Figure 2:**
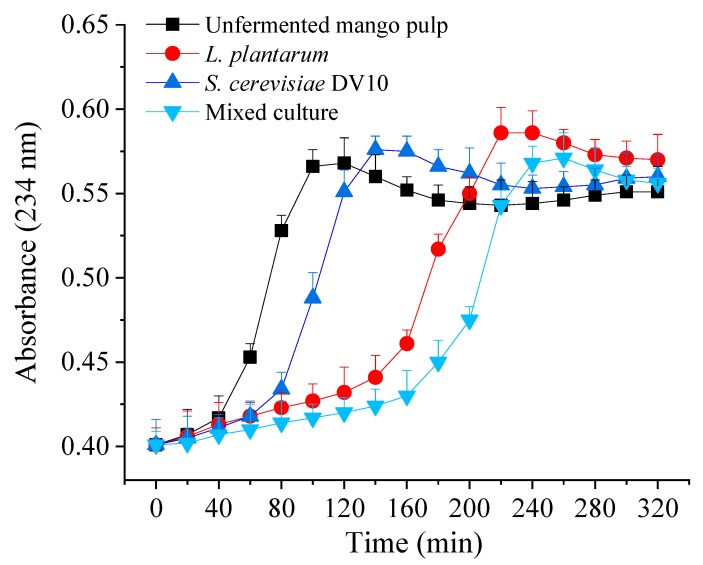
Effect of unfermented mango pulp and fermented mango pulp upon lag time of conjugated diene (CD) formation.

The effect of unfermented and different fermented mango pulp on copper-induced LDL oxidation kinetics is shown in Figure 2. Lipid peroxidation may cause inflammation, cancer, xenobiotic toxicity and peroxidative tissue damage during aging [20]. The high level of oxidized LDL cholesterol is a risk factor for atherosclerosis [21]. The lag time was 50 min for the unfermented mango pulp, and the lag times of the single *S. cerevisiae* DV10, single *L. plantarum* and mixed fermentation mango pulp were 80, 150 and 190 min, respectively. Previous studies have found that some LAB or yeast can inhibit LDL oxidation [22,23]. Therefore, fermentation of mango pulp with *L. plantarum* and *S. cerevisiae* DV10 can increase its antioxidant activities. Similarly, it can be seen that the co-cultivation of *S. cerevisiae* DV10 and *L. plantarum* had the longest lag time and the strongest LDL oxidation inhibition.

### 2.5. Changes in Organic Acids

**Figure 3:**
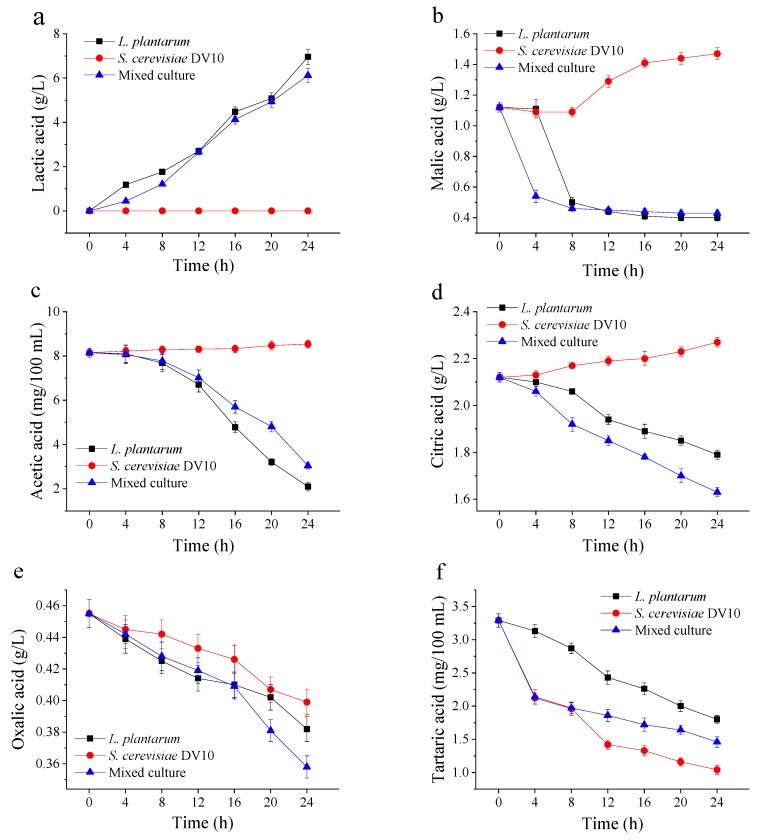
Organic acids evaluated during 24 h of mango slurry fermentation. (**a**) Lactic acid; (**b**) malic acid; (**c**) acetic acid; (**d**) citric acid; (**e**) oxalic acid and (**f**) tartaric acid.

The changes in malic, lactic, acetic, citric, oxalic and tartaric acids during 24 h fermentation of mango pulp were determined (Figure 3). Organic acids were present in the fermentation product because of hydrolysis and microbial activity. Lactic acid was the main metabolite produced in mango slurry fermentation with *L. plantarum*. As the LAB population increased, the lactic acid content increased. The lactic acid concentrations of single *L. plantarum* and co-cultured mango pulp were 6.96 g/L and 6.12 g/L at 24 h of fermentation, respectively. The malic acid content in unfermented mango slurry was 1.12 g/L, and it rapidly reduced in the first 8 h of the fermentation in the assay containing *L. plantarum* (single and co-culture) to 0.4 g/L. In the assay containing single *S. cerevisiae* DV10, the malic acid content slowly increased to 1.47 g/L during 24 h fermentation. High malic acid concentrations can have a negative effect on organoleptic properties of beverages [24]. The acetic acid concentrations of the single *L. plantarum* and co-cultured mango pulp decreased from 8.15 to 2.1 mg/100 mL and 3.04 mg/100 mL over 24 h, respectively. This may be due to consumption of acetic acid as a carbon source by *L. plantarum*. The decrease of acetic acid may be a positive factor, since this acid may provide an off-flavor in high concentrations [25].

In the assays containing *L. plantarum* (single and co-culture), the citric acid content of showed a decreasing trend. The citric acid content of the fermented mango pulp containing *L. plantarum* decreased from 2.12 to 1.79 g/L (single) and 1.63 g/L (co-culture) over 24 h. Citric acid can be metabolized by LAB to produce acetic, lactic acids and diacetyl. This metabolism has also been described in other studies of LAB and yeast co-cultivation [26]. After 24 h of fermentation, the oxalic and tartaric acid concentrations in the fermented mango pulp decreased. The decrease in tartaric acid concentration may have been related to tartrate precipitate formation. These organic acids may interact with other substances such as alcohols and aldehydes, producing other flavor compositions during the fermentation process [27].

### 2.6. Changes in Volatile Compounds

Volatile compounds were detected by using GC–MS for all fermentation assays after 24 h, as shown in Table 2. Terpenes were the main volatile substances in fresh mango pulp. In addition, some esters, acids, ketones and aldehydes were also important in the flavor of fresh mango pulp. Most of the volatiles found in mango pulp have been reported elsewhere [28]. Terpenes have a pungent aroma. The terpene content in the fermentation of *S. cerevisiae* DV10 (single and co-culture) significantly decreased over 24 h. This result was consistent with other reports [29]. 

*S. cerevisiae* DV10 (single and co-culture) produced more alcohol compounds than the assay with single *L. plantarum*. Yeast is an important alcohol producer with unique flavor characteristics that are additionally contributed to by the derived esters [30]. Yeast can produce a variety of volatile compositions, such as esters, alcohols, ketones and aldehydes, which have a positive impact on flavor and organoleptic properties of fermented foods. After 24 h of fermentation, there were some alcoholic substances with pleasant flavor characteristics. Phenylethyl alcohol emits a rose aroma; 3-methyl-1-butanol has a banana-like and pear-like aroma and 1-hexanol has a rich fruity and aromatic flavor [30]. In the present study, the 3-methyl-1-butanol content was highest in mango slurry inoculated with a single *S. cerevisiae* DV10. In another study it has fermented cassava with a combination of LAB and yeast and found higher 3-methyl-1-butanol concentrations in a single *S. cerevisiae* fermentation culture, consistent with our findings [31]. Phenylethanol was detected during all fermentation tests. The phenylethyl alcohol content of the *S. cerevisiae* DV10 (single and co-culture) fermentation was significantly higher than that of single *L. plantarum* culture. After 24 h of fermentation, *S. cerevisiae* DV10 (single and co-culture) produced more esters, such as ethyl acetate, ethyl octanoate, ethyl quinate and ethyl hexanoate, compared to the assay with single *L. plantarum*. These esters positively contribute to the overall quality of the fermented mango pulp and most produce moderate “floral” or “fruit” flavors [29].

The aldehydes content in the three cultures decreased over 24 h. *S. cerevisiae* DV10 (single and co-culture) fermented mango pulp showed a decreased ketone content and an increased alkane content. Compared to unfermented mango pulp, the content of butyric acid in single *L. plantarum* cultures slightly increased; in the *S. cerevisiae* DV10 (single and co-culture) cultures, the butyric acid content significantly (*p* < 0.05) decreased. Butyric acid has an unpleasant rancid butter odor and a spicy taste [31], therefore lower levels are desirable.

**Figure 4:**
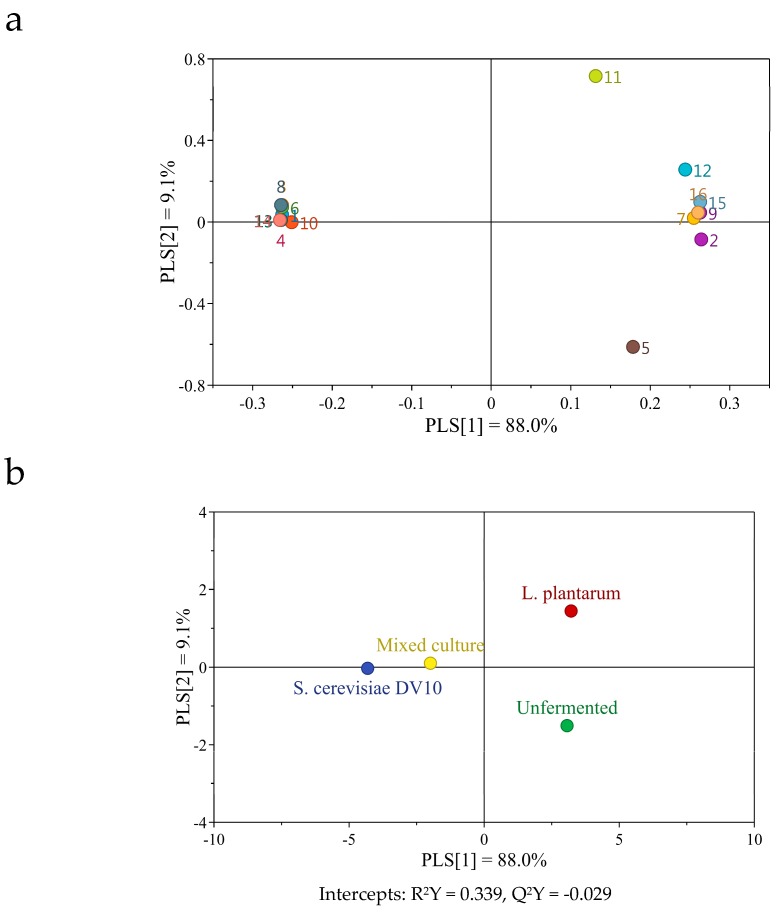
(**a**) Loadings scatter plot of PLS-DA of different constituents. 1, Phenylethyl Alcohol; 2, Phellandrene; 3, Octanoic acid, ethyl ester; 4, Hexanoic acid, ethyl ester; 5, Hexanoic acid; 6, Ethanol; 7, d-Limonene; 8, Decanoic acid, ethyl ester; 9, Butanoic acid; 10, Acetic acid, 2-phenylethyl ester; 11, Acetic acid; 12, 3-Penten-2-one; 13, Isoamyl acetate; 14, 3-methyl-1-Butanol; 15, 3-Carene; 16, (+)-4-Carene. (**b**) Scores scatter plot in different probiotics fermentation of mango slurry.

After 24 h of fermentation, partial least squares-discriminant analysis (PLS-DA) was performed to correlate volatile compounds with different fermentation measurements. Figure 4 showed the score plot of the volatile compounds, which contributed 97.1% of the total variance (PLS [1] + PLS [2]). The assay with *S. cerevisiae* DV10 (single and co-culture) were localized on negative semiaxis of PLS [1] owing to their high hexanoic acid, ethyl ester, acetic acid, 2-phenylethyl ester, phenylethyl alcohol, 3-methyl-1-butanol, isoamyl acetate, octanoic acid, ethyl ester and ethanol contents. The unfermented mango pulp and that fermented with a single *L. plantarum* were localized on positive semiaxis of PLS [1] mainly owing to their high phellandrene, d-limonene, 3-carene, butanoic acid, (+)-4-carene and 3-penten-2-one contents. Since the fermentation product of single *L. plantarum* contains more acetic acid, the unfermented mango pulp has a higher hexanoic acid content and can be differentiated from the other fermentation products by PLS [2]. These results showed that unfermented mango pulp and that fermented with a single *L. plantarum* had an unpleasant pungent odor. The assay with *S. cerevisiae* DV10 (single and co-culture) had a pleasant fruity and aromatic flavor.

## 3. Materials and Methods

### 3.1. Materials

Tainong mangoes were obtained from Hainan Dachuan Food Co., Ltd. (Hainan, China). *L. plantarum GIM1.140* was obtained from Guangdong Microbial Culture Center (Guangdong, China). The active dry yeast strain (*S. cerevisiae* DV10) was obtained from Lallemand Inc. (Montreal, Canada).

### 3.2. Fermented Mango Slurry

Mango peeled and added 10% water to beat. Its soluble solid content was increased to 21.6° Brix with sucrose. Next, mango slurry was heated for 10 min at 90 °C and cooled to 25 °C. Microbial cells were inoculated in the mango slurry with a population of 5 log CFU/mL for *S. cerevisiae* DV10 and 7 log CFU/mL for *L. plantarum* in both single and co-culture fermentations. Fermentation was conducted at 28 °C for 24 h, and the process was repeated three times.

### 3.3. Enumeration of Microorganisms

The total LAB and yeast populations were determined as previously described method [25]. The total LAB populations were determined by plating in the MRS agar (supplemented with 50 mg/L of nystatin), and plates were incubated at 37 °C for 48 h. The total yeast populations were determined by plating in YPD agar (supplemented with 50 mg of chlortetracycline and 100 mg of chloramphenicol), and plates were incubated at 30 °C for 48 h. The colony-forming units (CFU) were enumerated. The analyses were performed in triplicate.

### 3.4. The pH, Total Soluble Solids and Reducing Sugar Content

The pH value was determined by using a pH meter (FE20 pH meter). The Brix value was determined with a portable refractometer (ATAGO, Tokyo, Japan). The reducing sugar content was determined using the 3,5-dinitrosalicylic acid (DNS) method [32].

### 3.5. TPC

The TPC of the samples were measured in accordance with the Folin–Ciocalteu colorimetric method with several modifications [33]. A 0.3 mL sample was mixed with 0.2 mL of the Folin–Ciocalteu reagent and placed for 5 min. Next, 1.3 mL of 10% Na_2_CO_3_ solution was added, and the mixture was reacted 1.5 h in the dark. Samples absorbance at 765 nm was determined. Using gallic acid as the standard, the TPC of the samples were expressed as milligrams of gallic acid equivalents (GAEs).

### 3.6. Scavenging Effect on ABTS Radical

The sample was measured using the previously described method with minor modifications [34]. The 7.4 mM ABTS solution was added to 2.6 mM potassium persulfate solution, and kept in the dark at room temperature for 12 h. The ABTS radical solution was diluted with 10 mM PBS to an absorbance of 0.70 ± 0.02. A total of 3.9 mL of diluted ABTS radical solution was mixed with 0.1 mL of sample, and kept in the dark for 6 min. Absorbance at 734 nm was determined. The ABTS radical scavenging capacity was calculated using the following formula:

Radical scavenging capacity (%) = [(A_0_ − A_1_)/A_0_] × 100,

where A_0_ is the control (PBS) absorbance, and A_1_ is the extract absorbance.

### 3.7. Determination of FRAP

FRAP assay was measured following the previously described method with some improvements [16]. FRAP solution contained 0.5 mL of 20 mM FeCl_3_·6H_2_O, 0.5 mL of 10 M 2,4,6-tris(2-pyridyl)-s-triazine solution and 5 mL of 0.3 M acetate buffer. One mL of sample with 3 mL of the FRAP solution were mixed well, and stand at dark for 50 min. The absorbance at 593 nm was determined. Using FeSO_4_ as standard, and the final results were expressed as FeSO_4_ equivalents.

### 3.8. Copper-Induced LDL Oxidation

LDL oxidation was determined using a previously described method. The oxidation kinetics were determined by absorbance changes [23]. In this study, a 2-mL LDL was added to the sample (0.05% final concentration). The sample was placed at 37 °C, and was incubated with a CuSO_4_ solution for 15 min to initiate a peroxidation reaction.

### 3.9. Organic Acid Content

Fermented mango slurry organic acid contents were determined on a high-performance liquid chromatography (HPLC) system equipped with UV-visible detector and a ZORBAX SB-Aq column (4.6 mm × 250 mm, 5 µm; Agilent, Santa Clara, CA, USA). The column was eluted with the mobile phase (0.02 M ammonium dihydrogen phosphate: methanol = 97:3) at a flow rate of 0.8 mL/min at 30 °C [35].

### 3.10. Volatile Compound Content

The volatile compound content of the sample was measured as the previously described method [36], with minor modifications. Samples were collected for headspace extraction using an solid phase microextraction autosampler (Supelco, USA) for 30 min. Thermal desorption occurred in the injector port for 3 min at 230 °C. Separation was carried out on a Ptx–Wax capillary column of 30 m × 0.25 mm. The carrier gas He at 1 mL/min, and the temperature was set at 40 °C for 2 min, ramp of 5 °C/min up to 160 °C, and finally to 230 °C at 10 °C/min. Using the GC/MS solution software to collect data. The eluted volatile compositions were matched with the NIST 115 libraries via mass spectrometry and confirmed by linear retention index values.

### 3.11. Statistical Analysis

All the experiments were conducted in triplicate and data were reported as mean ±SD. Analysis of variance and significant difference tests were performed to identify differences among means by one-way ANOVA using SPSS software (version 16.0, Chicago, IL, USA).

## 4. Conclusions

This study showed the possibility of using a combination of *S. cerevisiae* DV10 and *L. plantarum* as starter cultures during mango slurry fermentation. The co-cultures of *S. cerevisiae* DV10 and *L. plantarum* could increase the product’s antioxidant activity. In addition, a co-culture with the *L. plantarum* and *S. cerevisiae* DV10 could decrease terpenes and produced various volatile compounds (alcohols and esters) that might improve the aromatic profiles of the fermented mango slurry. The sensory properties of the products should be evaluated at the next step. Further studies regarding the viability and benefits of these strains in the gut after consumption also need to be conducted.

## Figures and Tables

**Table 1:** Quality parameters, total phenolic content (TPC) and antioxidant capacity of mango slurry before and after 24 h fermentation.

Samples	pH	TSS (° Brix)	Reducing sugars (g/L)	TPC (mg GAE/100 mL)	ABTS (% Inh)	FRAP (mM FeSO_4_)
Unfermented	4.12 ± 0.15b	21.6 ± 0.5b	2.24 ± 0.07b	75.87 ± 1.43a	10.43 ± 0.25a	1.11 ± 0.03a
*L. plantarum*	3.54 ± 0.09a	21.2 ± 0.4b	2.13 ± 0.06b	86.59 ± 1.29c	15.29 ± 0.31c	1.47 ± 0.04b
*S. cerevisiae* DV10	3.98 ± 0.12b	20.2 ± 0.5a	1.97 ± 0.04a	79.41 ± 1.65b	12.72 ± 0.29b	1.16 ± 0.02a
Co-culture	3.55 ± 0.07a	20.3 ± 0.4a	2.00 ± 0.05a	89.25 ± 1.06d	16.11 ± 0.34d	1.49 ± 0.05b

Data represent the mean ± SD; values with different letters (a–d) in the same column are significantly different at *p* < 0.05; TSS, total soluble solids; TPC, total phenolic content; Inh, inhibition.

**Table 2:** Major volatile compounds (peak area × 10^6^) in mango slurry before and after 24 h fermentation.

Volatile Compounds	RT	RI	Unfermented Mango Slurry	*L. plantarum*	*S. cerevisiae* DV10	Co-Culture
Terpenes						
(+)-4-Carene	10.228	919	517.02 ± 25.85a	535.5 ± 26.78a	263.9 ± 18.2b	275.62 ± 18.78b
3-Carene	7.312	948	33.94 ± 1.7b	37.67 ± 1.88a	16.26 ± 0.81d	19.59 ± 0.98c
d-Limonene	8.434	1018	13.03 ± 0.65a	13.53 ± 0.68a	7.07 ± 0.35b	6.67 ± 0.33b
γ-Terpinene	9.452	998	2.83 ± 0.14a	2.83 ± 0.14a	1.62 ± 0.08c	2.32 ± 0.12b
o-Cymene	9.888	1042	5.84 ± 0.29a	5.76 ± 0.29a	2.93 ± 0.15c	4.04 ± 0.2b
β-Myrcene	7.796	958	8.28 ± 0.41a	12.93 ± 0.65b	5.25 ± 0.26c	5.15 ± 0.26c
Phellandrene	7.685	969	16.06 ± 0.8a	15.15 ± 0.76a	6.67 ± 0.33c	8.79 ± 0.44b
β-Ocimene	9.65	976	3.33 ± 0.17b	4.04 ± 0.2a	–	1.62 ± 0.08c
Caryophyllene	15.637	1494	1.21 ± 0.06a	–	–	–
α-Pinene	4.411	948	1.82 ± 0.09a	1.92 ± 0.1a	–	–
α-Copaene	14.05	1221	1.11 ± 0.06a	–	–	0.61 ± 0.03b
Subtotal			604.47	629.33	303.7	324.41
Alcohols						
Ethanol	2.829	463	–	22.56 ± 1.13c	248.22 ± 12.41a	141.84 ± 7.09b
2-Penten-1-ol	10.871	769	9.22 ± 0.46a	1.65 ± 0.08b	–	0.4 ± 0.02c
1-Hexanol	11.52	860	0.54 ± 0.03c	1.35 ± 0.07a	–	0.64 ± 0.03b
(Z)-3-Hexen-1-ol	12.062	868	–	1.58 ± 0.08a	–	0.3 ± 0.02b
1-Octanol	14.979	1059	0.64 ± 0.03b	1.68 ± 0.08a	–	1.78 ± 0.09a
(E,Z)-3,6-Nonadien-1-ol	17.564	1175	1.68 ± 0.08a	1.82 ± 0.09a	0.57 ± 0.03c	1.01 ± 0.05b
3-methyl-1-Butanol	8.72	697	–	–	89.18 ± 4.46a	52.18 ± 2.61b
Phenylethyl Alcohol	19.359	1136	–	1.92 ± 0.1c	53.6 ± 2.68a	29.36 ± 1.47b
2-methyl-1-Propanol	6.289	597	–	–	14.85 ± 0.74a	6.13 ± 0.31b
cis-p-Mentha-2,8-dien-1-ol	18.55	1140	–	7.37 ± 0.37a	1.65 ± 0.08c	4.38 ± 0.22b
[R-(R*,R*)]-2,3-Butanediol	14.564	743	–	–	4.41 ± 0.22a	1.14 ± 0.06b
3,7-dimethyl-1,6-Octadien-3-ol	14.835	1082	0.77 ± 0.04b	1.11 ± 0.06a	0.57 ± 0.03d	0.67 ± 0.03c
α-Terpineol	16.889	1143	0.54 ± 0.03a	0.57 ± 0.03a	–	–
Benzyl alcohol	18.957	1036	–	1.38 ± 0.07a	–	0.84 ± 0.04b
2,4-bis(1,1-dimethylethyl)-Phenol	23.362	1555	4.48 ± 0.22a	1.52 ± 0.08c	2.93 ± 0.15b	1.11 ± 0.06d
6-Nonen-1-ol	17.155	1167	–	–	2.26 ± 0.11a	2.42 ± 0.12a
Eugenol	21.995	1392	–	1.18 ± 0.06b	–	0.84 ± 0.04c
Subtotal			17.88	45.69	418.24	245.06
Esters						
Ethyl Acetate	2.35	586	33.03 ± 1.65a	5.76 ± 0.29d	22.12 ± 1.11b	9.49 ± 0.47c
Octanoic acid, ethyl ester	13.079	1183	5.05 ± 0.25d	37.64 ± 1.88c	317.14 ± 15.86a	193.22 ± 9.66b
Butanoic acid, ethyl ester	4.816	785	7.17 ± 0.36b	7.17 ± 0.36b	8.59 ± 0.43a	4.14 ± 0.21c
Decanoic acid, ethyl ester	16.239	1381	2.22 ± 0.11d	13.54 ± 0.68c	116.55 ± 5.83a	77.17 ± 3.86b
Hexanoic acid, ethyl ester	9.265	984	–	–	77.87 ± 3.89a	43.43 ± 2.17b
Dodecanoic acid, ethyl ester	18.801	1580	1.72 ± 0.09c	–	15.35 ± 0.77a	4.04 ± 0.2b
Isoamyl acetate	6.779	820	–	–	28.58 ± 1.43a	16.36 ± 0.82b
Acetic acid,2-phenylethyl ester	18.348	1259	–	–	27.98 ± 1.4a	9.8 ± 0.49b
Octanoic acid, methyl ester	12.267	1083	–	–	5.86 ± 0.29a	5.96 ± 0.3a
Ethyl 9-decenoate	16.913	1371	–	–	20.81 ± 1.04a	–
Tetradecanoic acid, ethyl ester	21.017	1779	–	–	4.55 ± 0.23a	0.61 ± 0.03b
Decanoic acid, methyl ester	15.618	1282	–	–	5.45 ± 0.27a	2.93 ± 0.15b
Hexanoic acid, methyl ester	8.229	884	–	1.21 ± 0.06b	1.82 ± 0.06a	1.82 ± 0.09a
4-Terpinenyl acetate	6.901	1327	–	–	–	2.83 ± 0.14a
Formic acid, butyl ester	7.261	783	1.62 ± 0.08b	2.02 ± 0.11a	–	–
(S)-1-Alanine ethylamide	1.283	864	–	2.53 ± 0.12b	–	2.83 ± 0.15a
Formic acid, heptyl ester	13.33	1081	–	2.42 ± 0.12a	–	–
Subtotal			50.81	72.29	652.67	374.63
Acids						
Butanoic acid	15.839	775	34.64 ± 1.53b	37.02 ± 1.85a	14.04 ± 0.7d	16.82 ± 0.84c
Hexanoic acid	23.507	974	64.99 ± 3.25a	22.67 ± 1.13b	13.58 ± 0.68d	16.51 ± 0.83c
Acetic acid	13.037	576	–	55.2 ± 2.76a	–	17.07 ± 0.85b
Octanoic acid	20.917	1173	2.88 ± 0.14d	4.34 ± 0.22c	20.91 ± 1.05a	15.91 ± 0.8b
n-Decanoic acid	22.978	1372	0.91 ± 0.05c	0.4 ± 0.02d	13.79 ± 0.69a	4.75 ± 0.24b
Octadecanoic acid	24.14	2167	3.23 ± 0.16c	17.57 ± 0.88b	27.72 ± 1.39a	1.46 ± 0.07d
Dodecanoic acid	24.845	1570	1.31 ± 0.07d	3.43 ± 0.17a	1.72 ± 0.09c	3.08 ± 0.15b
n-Hexadecanoic acid	24.718	1968	–	9.04 ± 0.45a	–	2.53 ± 0.13b
Subtotal			107.96	149.67	91.76	78.13
Aldehydes						
(E,Z)-2,6-Nonadienal	15.366	1120	9.29 ± 0.46a	–	–	–
Furfural	13.331	831	3.74 ± 0.19a	–	–	–
(E,E)-2,4-Heptadienal	13.865	921	4.24 ± 0.21a	–	–	–
Nonanal	12.286	1104	3.74 ± 0.19a	–	–	–
Citral	16.762	1174	7.78 ± 0.39b	10.5 ± 0.53a	–	11.51 ± 0.58a
2-Hexenal	8.754	814	2.63 ± 0.13a	–	–	–
Subtotal			31.42	10.5	0	11.51
Ketones						
3-Penten-2-one	6.686	662	17.17 ± 0.86b	28.89 ± 1.44a	–	–
4-hydroxy-2-Pentanone	13.22	817	10.2 ± 0.51a	9.7 ± 0.48a	3.13 ± 0.16b	3.03 ± 0.15b
5-ethyldihydro-2(3*H*)-Furanone	16.831	986	10.4 ± 0.52a	7.68 ± 0.38b	2.83 ± 0.14d	4.75 ± 0.24c
5-butyldihydro-2(3*H*)-Furanone	19.425	1184	3.54 ± 0.18a	1.62 ± 0.08b	–	–
trans-β-Ionone	19.765	1457	3.03 ± 0.15a	1.01 ± 0.05d	2.32 ± 0.12b	1.82 ± 0.09c
2-Heptanone	8.068	853	–	4.55 ± 0.23a	–	1.01 ± 0.05b
Acetoin	10.085	717	–	19.38 ± 0.97a	9.59 ± 0.48c	16.16 ± 0.81b
2,3-Butanedione	3.523	691	–	6.16 ± 0.31a	–	–
1-(3-methylphenyl)-Ethanone	17.797	1142	5.56 ± 0.28a	–	–	–
4-methyl-4-Hexen-3-one	6.761	838	4.55 ± 0.23a	–	–	–
tetrahydro-6-methyl-2*H*-Pyran-2-one	17.936	1006	13.94 ± 0.7c	8.32 ± 0.42d	16.78 ± 0.64a	15.08 ± 0.75b
Subtotal			68.39	87.31	34.65	41.85
Alkanes						
(2-methyl-1-propenyl)-Benzene	12.993	1077	14.04 ± 0.7a	12.63 ± 0.63ab	11.92 ± 0.6b	10.2 ± 0.51c
1,3,8-p-Menthatriene	12.243	1029	–	2.32 ± 0.12a	–	–
bis(1-methylethylidene)-Cyclobutene	12.81	983	2.63 ± 0.13a	2.02 ± 0.1b	1.52 ± 0.08c	1.01 ± 0.05d
Styrene	9.588	883	–	–	11.41 ± 0.57a	2.12 ± 0.11b
2,6,10,14-tetramethyl-Pentadecane	20.42	1653	–	–	11.51 ± 0.58a	7.07 ± 0.35b
Heneicosane	18.106	2109	12.83 ± 0.64b	10.91 ± 0.55c	20.5 ± 1.03a	18.28 ± 0.91a
2-methyloctacosane	18.397	2840	2.32 ± 0.12a	–	–	–
Subtotal			31.82	27.88	56.86	38.68
Others						
trans-2-(2-Pentenyl)furan	10.603	1048	1.82 ± 0.09b	2.43 ± 0.09a	–	0.91 ± 0.05c
1,1-diethoxy-Ethane	2.439	705	–	–	13.23 ± 0.66a	6.77 ± 0.34b
2,3-dihydro-Benzofuran	23.969	1036	–	3.74 ± 0.19a	0.81 ± 0.03b	0.81 ± 0.04b
2,4,5-trimethyl-1,3-Dioxolane	3.022	761	–	–	12.02 ± 0.47b	13.53 ± 0.38a
Subtotal			1.82	6.17	26.06	22.02

Values are expressed as the mean ± SD. Values with different letters (a–d) in the same row are significantly different at *p* < 0.05. RT, retention time; RI, retention index; PA, peak area; “–“, not detected.
